# Degradation of Bisphenol A in an Aqueous Solution by a Photo-Fenton-Like Process Using a UV KrCl Excilamp

**DOI:** 10.3390/ijerph18031152

**Published:** 2021-01-28

**Authors:** Denis Aseev, Agniya Batoeva, Marina Sizykh, Daniil Olennikov, Galina Matafonova

**Affiliations:** 1 Baikal Institute of Nature Management of Siberian Branch of Russian Academy of Sciences, 6, Sakhyanovoy St., 670047 Ulan-Ude, Russia; aseev.denis.g@gmail.com (D.A.); abat@binm.ru (A.B.); marisyz@binm.ru (M.S.); 2 Institute of General and Experimental Biology of Siberian Branch of Russian Academy of Sciences, 6, Sakhyanovoy St., 670047 Ulan-Ude, Russia; olennikovdn@mail.ru

**Keywords:** Bisphenol A, degradation, UV excilamp, persulfate, hydroxyl radicals, sulfate anion radicals

## Abstract

Bisphenol A (BPA), a precursor to important plastics, is regarded as a common aquatic micropollutant with endocrine-disrupting activity. In the present study, we explored the capability of a UV KrCl excilamp (222 nm) to degrade BPA by a photo-Fenton-like process using persulfate under flow-through conditions. The first-order rate constants of degradation were obtained and the mineralization of dissolved organic carbon (DOC) was estimated. The results showed complete BPA degradation and high DOC mineralization (70–97%). A comparative analysis of degradation rates and DOC removal in the examined systems (UV, Fe^2+^/S_2_O_8_^2−^, UV/S_2_O_8_^2−^ and UV/Fe^2+^/S_2_O_8_^2−^) revealed a significant synergistic effect in the photo-Fenton-like system (UV/Fe^2+^/S_2_O_8_^2−^) without the accumulation of toxic intermediates. This indicated that the BPA was oxidized via the conjugated radical chain mechanism, which was based on the photo-induced and catalytic processes involving HO^•^ and SO_4_^−•^ radicals. The primary intermediates of BPA degradation in the UV/Fe^2+^/S_2_O_8_^2−^ system were identified by HPLC/MS and the oxidation pathway was proposed. The high performance of the photo-Fenton-like process employing a quasi-monochromatic UV radiation of a KrCl excilamp offers promising potential for an efficient removal of such micropollutants from aqueous media.

## 1. Introduction

Over the last few decades, rapid industrial development has produced a great number of hazardous biorecalcitrant micropollutants, which are discharged into the aquatic ecosystems with insufficiently treated wastewater effluents. Among these, Bisphenol A (BPA), a precursor to polycarbonate plastics and similar products, is regarded as a common aquatic micropollutant with endocrine-disrupting activity [1,2]. BPA can be readily removed in water via oxidation with hydroxyl (HO^•^) and sulfate anion (SO_4_^•−^) radicals generated in advanced oxidation processes (AOPs).

To date, sulfate radical-based AOPs have emerged as an advanced technology for water and wastewater treatment [3,4]. Sulfate anion radicals have many advantages over HO^•^ such as a higher red-ox potential, a longer half-life and a broader range of operating conditions [5]. They can be generated upon the activation of persulfates with heat, ultraviolet (UV) radiation, ultrasound, microwave radiation or in the presence of transition metals [6,7,8]. Persulfates are widely applied as a SO_4_^•−^ source owing to high water solubility, availability, easy transportation, storage and dosage [7,8].

Photo-activated persulfate-based AOPs have been intensively developed over the last years and recognized as a highly efficient method of pollutant degradation and a cost-effective way of up-scaling the water treatment [5,9]. Apart from activating persulfate directly, UV irradiation also enhances a Fenton-like process involving persulfate and ferrous ions. Photo-Fenton-like processes, utilizing conventional UV mercury lamps, have been proven to attain an enhanced degradation of organic micropollutants [10,11,12]. Narrow band mercury-free UV sources such as excilamps have a high potential for improving oxidation efficiency [13,14,15,16]. Specifically, previous studies with UV excilamps reported the complete degradation of organic pollutants by photo-Fenton-like and UV/H_2_O_2_ processes using a KrCl excilamp (222 nm) (Table 1).

However, there are only two reports on pollutant degradation by a photo-Fenton-like process using a UV excilamp and persulfate [17,19], which focused on obtaining kinetic data without elucidating the oxidation pathway and the toxicity changes. The objective of this study was to establish the kinetics of BPA degradation and mineralization as well as its oxidation pathway and detoxification under exposure to KrCl excilamp irradiation in the presence of persulfate and ferrous ions (II). To the best of our knowledge, this is the first application of a UV KrCl excilamp for activating persulfate in the photo-Fenton-like system (UV/Fe^2+^/S_2_O_8_^2−^) to degrade, mineralize and detoxify BPA and its products under flow-through conditions.

## 2. Materials and Methods

### 2.1. Chemicals

Aqueous solutions of BPA (4,4’-dihydroxy-2,2-diphenylpropane, ≥99%, Sigma-Aldrich, St. Louis, MI, USA) were prepared in distilled water (χ < 2 μS/cm) at a concentration of 43.8 μM (10 mg/L) and at an unadjusted pH of 5.5. Iron (II) sulfate (FeSO_4_ × 7H_2_O, ≥ 99.5%, Scharlab S.L., Barcelona, Spain) and potassium persulfate (K_2_S_2_O_8_, 99%, Khimreaktivsnab, Ufa, Russia) were used as received.

### 2.2. Experimental Procedure

**Figure 1:**
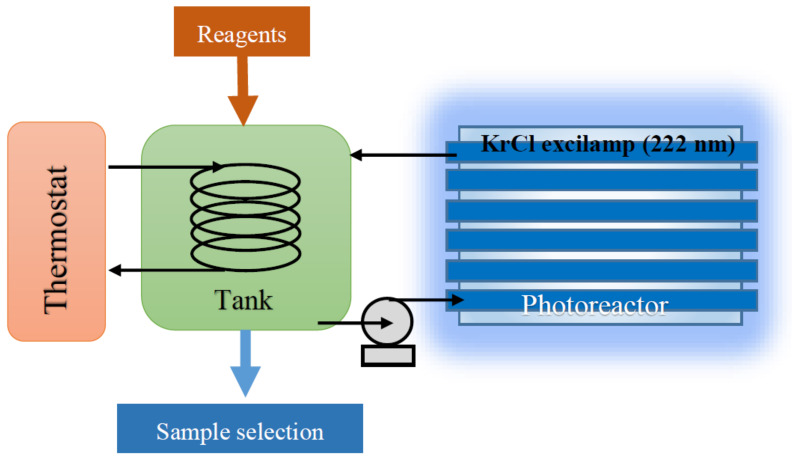
Schematic representation of the experimental setup.

Experiments were performed in a flow-through photoreactor at a rate of 0.5 L/min. The experimental setup consisted of a thermostatic tank (V = 0.4 L, T = 25 °C), a laboratory peristaltic pump and a tubular photoreactor (Figure 1). The photoreactor comprised a KrCl excilamp (222 nm, 23 W, “Excilamp” Ltd., Tomsk, Russia), a reflector made of aluminum sheet and quartz tubes with circulating water. The UV irradiance, determined by atrazine actinometry [27], was 0.95 mW/cm^2^.

The BPA concentration during treatment was monitored by HPLC using an Agilent 1260 Infinity liquid chromatograph with a fluorescence detector, 1260 FLD (λ_ex/em_ = 230/315 nm). An isocratic elution was performed on a Zorbax SB-C18 column with a particle size of 5 μm. A mixture of acetonitrile with 75 mM acetic acid at a volume ratio of 45:55 was used as the mobile phase; the elution rate was 0.5 mL/min, the column thermostat temperature was set at 35 °C and the injection volume was 70 μL. The samples were prepared by microfiltration using PTFE 0.45 µm membrane filters (Vladisart, Vladimir, Russia).

The BPA intermediates were identified by RP-HPLC-DAD-ESI-MS using a LCMS 8050 liquid chromatograph (Shimadzu, Columbia, MD, USA) coupled with a diode-array detector and a triple-quadrupole detector with electrospray ionization. Chromatographic separation was performed on a GLC Mastro C18 column (150 × 2.1 mm^2^, Ø 3 μm; Shimadzu, Kyoto, Japan) at a column thermostat temperature of 30 °C. Solvents A and B (water and acetonitrile) were gradually eluted for 2 min to reach 70–100% B. The injection volume was 1 μL and the elution rate was 200 μL/min. For ESI-MS, the parameters were set as follows. The temperature levels of the ESI interface, the desolvation line and the heat block were 300 °C, 250 °C and 400 °C, respectively. The flow levels of the nebulizing gas (N_2_), the heating gas (air) and the collision-induced dissociation gas (Ar) were 3 L/min, 10 L/min and 0.3 mL/min, respectively. The capillary voltage was kept at −4.0 kV in the negative mode. ESI-MS spectra were recorded by scanning in the range of m/z 100–1900.

The mineralization was monitored by dissolved organic carbon (DOC) removal, as determined on a Shimadzu TOC-L CSN analyzer (detection limit 50 μg/L).

The degradation efficiency and DOC removal were calculated by Equation (1):

E(%) = 100 (C_0_ − C_τ_)/C_0_ (1)

where C_0_ and C_τ_ were the initial and residual concentrations of BPA (DOC) at a time τ (min), respectively.

All experiments were performed in duplicate.

The toxicity of the treated solution was evaluated by bioluminescent assay [28], which was based on measuring the bioluminescence inhibition of the recombinant strain of *E. coli* K12 TG1 carrying lux operons of the luminescent *Photobacterium leiognathi*, using a luminometer Biotox-10 (Nera-S Ltd., Moscow, Russia). The toxicity index (T) after biosensor exposure to the sample for 30 min was determined by Equation (2):

T = 100 (I_k_ − I)/I_k_ (2)

where I_k_ and I were the luminescence intensity (impulse/s) of a control and a sample, respectively. The intensity values were recorded in triplicate. The technique admitted three threshold levels of toxicity index: T < 20 (non-toxic), 20 ≤ T < 50 (toxic) and T ≥ 50 (highly toxic).

## 3. Results

### 3.1. Degradation by Persulfate Activated With KrCl Excilamp (UV/S_2_O_8_^2−^)

Direct UV irradiation without persulfate (direct photolysis) showed a relatively low BPA degradation rate so that it was converted by 84% after 60 min treatment whereas the DOC removal reached only 13% after 4 h of exposure. No degradation was observed in the presence of persulfate without any UV exposure (data not shown).

**Figure 2:**
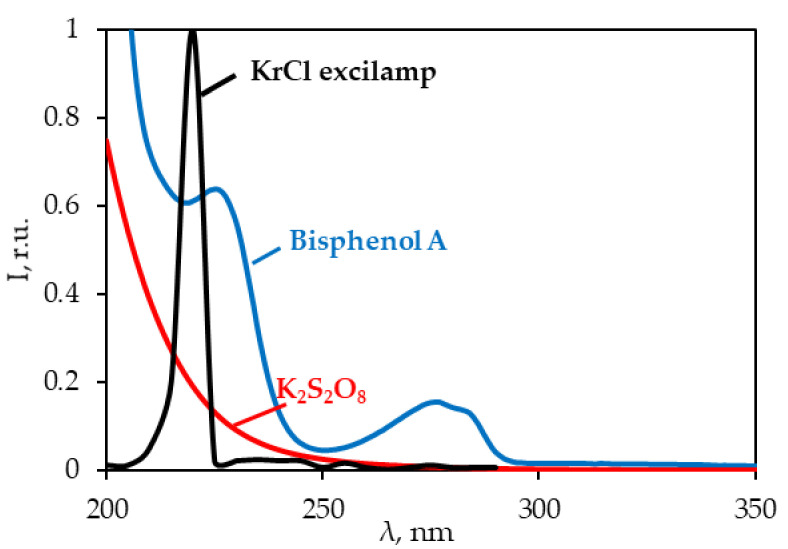
Absorption spectra of BPA and K_2_S_2_O_8_ and the emission spectrum of the KrCl excilamp.

An analysis of overlapping spectral absorption bands of potassium persulfate, BPA and the emission spectrum of the KrCl excilamp indicated the fundamental possibility of applying this UV source for persulfate-based AOPs (Figure 2).

**Figure 3:**
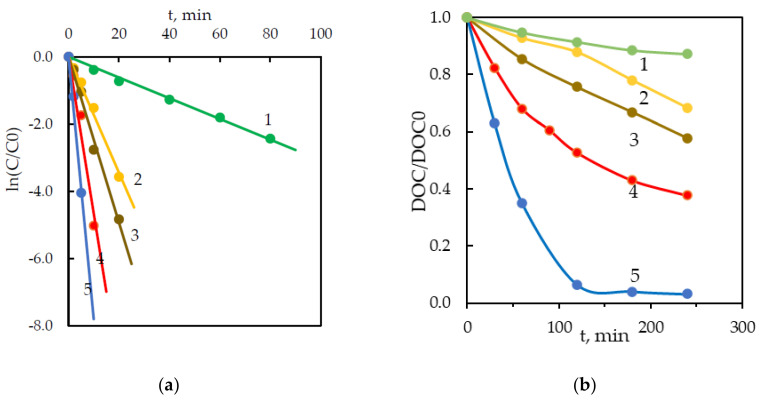
Kinetic curves for BPA degradation (**a**) and mineralization (**b**) in the UV/S_2_O_8_^2−^ system. [S_2_O_8_^2−^]/[BPA] (M/M) = 0 (curve 1), 3 (curve 2), 5 (curve 3), 10 (curve 4), 20 (curve 5). [BPA]_0_ = 43.8 µM, pH_0_ = 5.7.

Indeed, the addition of persulfate rapidly increased the degradation rate even at a low molar ratio of [S_2_O_8_^2−^]:[BPA] = 3:1 (Figure 3a). In the UV/S_2_O_8_^2−^ system, the apparent first-order rate constant of BPA degradation was 5.7 times higher than that observed under direct UV photolysis (Table 2). A further increase in the molar ratio to 20:1 also substantially increased the rate constants (4.5 times) and reduced the total treatment time for full BPA elimination from 30 to 10 min. Moreover, a higher mineralization was attained due to the advanced transformation of the intermediates.

Persulfate and BPA in a molar ratio of 20:1 corresponded to 56% of the stoichiometrically required amount (36 mol S_2_O_8_^2−^ per 1 mol BPA), according to the hypothetical equation of complete mineralization (3):

C_15_H_16_O_2_ + 72SO_4_^•−^ + 28H_2_O → 15CO_2_ + 72SO_4_^2−^ + 72H^+^. (3)

It was found that BPA at this molar ratio was completely degraded after 10 min treatment. The highest percentage of DOC removal (97%) was obtained after 2 h of exposure (Figure 3b).

Sharma et al. [29] studied the kinetics of BPA degradation by a UV/S_2_O_8_^2−^ process using a low-pressure mercury lamp (254 nm). The reported rate constant (0.0118 min^–1^) under similar experimental conditions ([BPA]_0_ = 40 μM, [S_2_O_8_^2−^]:[BPA] = 31.5 (M/M)) was significantly lower than that obtained with the KrCl excilamp (0.78 min^−1^). This could be primarily attributed to the increased SO_4_^−^^•^ generation at 222 nm due to the higher absorbance (by one order of magnitude) of persulfate compared with 254 nm (Figure 2). A higher ^•^OH exposure with the KrCl excilamp was also reported earlier under the UV/H_2_O_2_ scheme [24].

In the UV/S_2_O_8_^2−^ system, persulfate was photoactivated via the homolytic cleavage of the -O-O- bond to form SO_4_^−•^ (reaction 4). In the reaction of SO_4_^−•^ with H_2_O or with OH^−^ ions under alkaline conditions HO^•^ could be also formed [30]:

S_2_O_8_^2−^ + *hν* → 2SO_4_^•−^ (4)

SO_4_**^∙^**^−^ + H_2_O → SO_4_^2−^ + H^+^ + HO^•^  *k* = 6.6 × 10^2^ M^−1^s^−1^ (5)

SO_4_**^∙^**^−^ + OH^−^ → SO_4_^2−^ + HO^•^     *k* = 7 × 10^7^ M^−1^s^−1^. (6)

However, along with reactions 7 and 8 at high rates [31], the contribution of direct photolysis should be also taken into account (reaction 9).

BPA + SO_4_^•−^ → intermediatesV     *k* = 4.7 × 10^9^ M^−1^ s^−1^ (7)

BPA + HO^•^ → intermediates     *k* = 8.8 × 10^9^ M^−1^ s^−1^ (8)

BPA + *hν* → intermediates. (9)

Finally, BPA was completely degraded and a high DOC mineralization was attained by UV-activated persulfate at a molar ratio of [S_2_O_8_^2−^]:[BPA] = 20:1. To enhance the degradation efficiency and reduce persulfate consumption, we further examined the performance of the photo-Fenton-like process (UV/Fe^2+^/S_2_O_8_^2−^) where persulfate was activated simultaneously with UV radiation and Fe^2+^ ions.

### 3.2. Degradation by a Photo-Fenton-Like Process (UV/Fe^2+^/S_2_O_8_^2−^)

**Figure 4:**
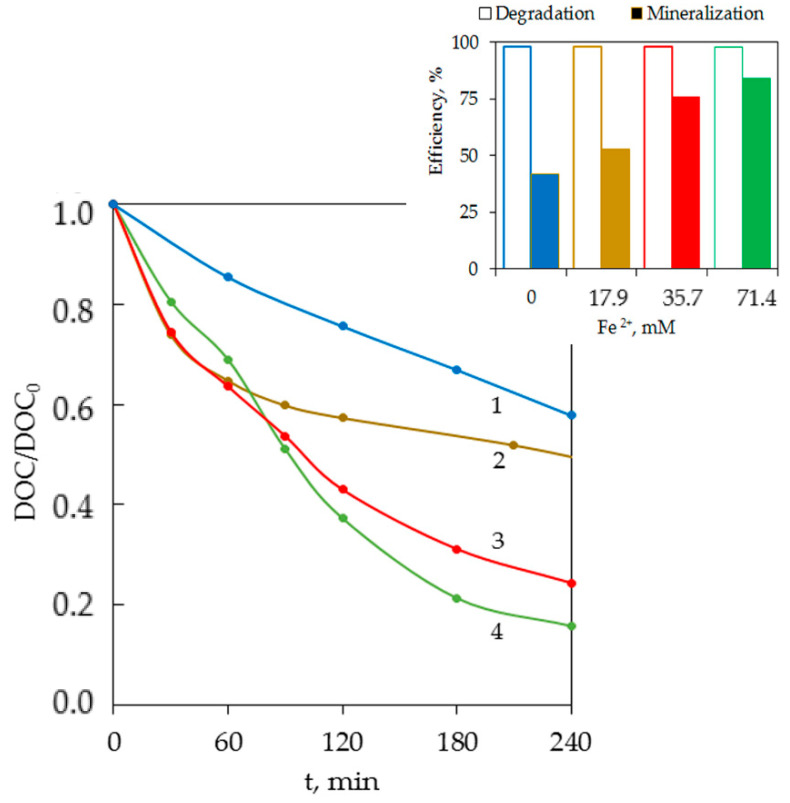
Influence of Fe^2+^ concentration on DOC mineralization in the UV/Fe^2+^/S_2_O_8_^2−^ system: [BPA]_0_ = 43.8 μM, [S_2_O_8_^2−^]:[BPA] = 5:1. Fe^2+^ concentration (μM): 0 (curve 1), 17.9 (curve 2), 35.7 (curve 3), 71.4 (curve 4). The inset shows BPA degradation (t = 20 min) and DOC mineralization (t = 240 min) as a function of the Fe^2+^ concentration.

To obtain the kinetic patterns of BPA degradation in the photo-Fenton-like system and compare the effectiveness of different oxidation systems, the experimental series was performed at a molar ratio of [S_2_O_8_^2−^]:[BPA] = 5:1. The effect of different Fe^2+^ concentrations on BPA and DOC elimination was studied in the range of 17.9–71.4 μM (1–4 mg/L). As shown in Figure 4, the Fe^2+^ concentration significantly influenced the DOC mineralization. Specifically, on increasing it from 17.9 to 71.4 μM, the mineralization was improved from 53 to 84% after 4 h of exposure.

It is known that Fe^2+^ and Fe^3+^ ions catalyze the persulfate decomposition with the formation of SO_4_^•^^−^ [32,33,34]:

Fe^2+^ + S_2_O_8_^2−^ → SO_4_^•−^ + SO_4_^2−^ + Fe^3+^ (10)

Fe^3+^ + S_2_O_8_^2−^ → 2SO_4_^•−^ + Fe^2+^. (11)

Additionally, the photoreduction of Fe^3+^ ions from aqua and intermediate complexes is accompanied by formation of hydroxyl and substrate radicals [33]:

(12) $${\mathrm{FeOH}}^{2+}\;\overset{h\nu }{\to }\;{\mathrm{Fe}}^{2+}\;+\;{\mathrm{HO}}^{•}$$

(13) $${\mathrm{LFe}}^{3+}\;\overset{h\nu }{\to }\;{\mathrm{Fe}}^{2+}\;+\;L^{•}.$$

**Figure 5:**
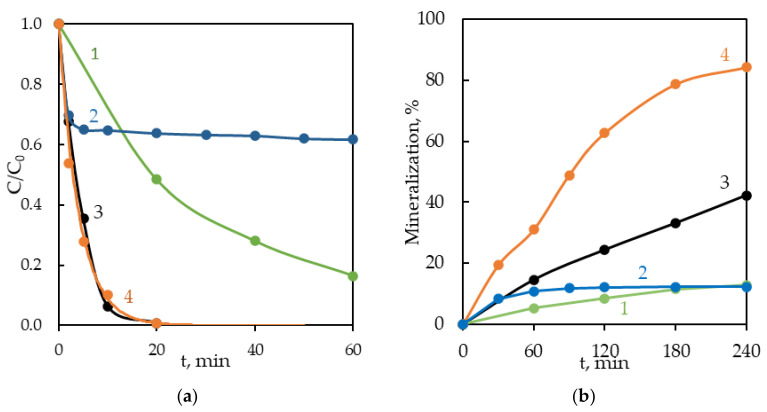
Kinetic curves of BPA degradation (**a**) and mineralization (**b**) in different oxidation systems: UV (curve 1), Fe^2+^/S_2_O_8_^2^^−^ (curve 2), UV/S_2_O_8_^2^^−^ (curve 3), UV/Fe^2+^/S_2_O_8_^2^^−^ (curve 4). [BPA]_0_ = 43.8 μM, [Fe^2 +^]_0_ = 71.4 μM, [S_2_O_8_^2^^−^]:[BPA] = 5:1.

Contrary to expectations, the BPA degradation rates in the UV/Fe^2+^/S_2_O_8_^2^^−^ and UV/S_2_O_8_^2^^−^ systems were comparable (Figure 5a, curves 3 and 4). However, upon simultaneous activation of persulfate by Fe^2+^ and UV, the intermediates were oxidized more completely as evidenced by higher DOC mineralization (2 times after 4 h of exposure) (Figure 5b).

A comparative assessment of the effectiveness (E) of BPA degradation and DOC mineralization in different systems (UV, Fe^2+^/S_2_O_8_^2^^−^, UV/S_2_O_8_^2^^−^ and UV/Fe^2+^/S_2_O_8_^2^^−^) revealed a significant synergistic effect in the photo-Fenton-like system, UV/Fe^2+^/S_2_O_8_^2^^−^ (Figure 5). It was confirmed by a high synergistic index (φ = 1.55) for mineralization efficiency (%), which was calculated by the following Formula (14):

(14) $$\varphi =\;\frac{E\;{\left(\mathrm{UV}/{\mathrm{Fe}}^{2+}/S_{2}O_{8}{}^{2-}\right)}_{\;}}{E\;({\mathrm{Fe}}^{2+}/S_{2}O_{8}{}^{2-})+\;E{\left(\mathrm{UV}/S_{2}O_{8}{}^{2-}\right)}_{\;}}.$$

Along with the mineralization efficiency, the toxicity of the treated solutions was evaluated. As reported previously, the acute toxicity of the treated solutions could be increased due to formation of more toxic reaction products [35,36]. The BPA solution was moderately toxic before treatment (T = 20.4 ± 8.5). Subsequent treatment in the photo-Fenton-like system reduced the toxicity to 9.9 ± 3.7 after 5 min and fully detoxified the solution after 30 min (Table 3). This implied that no toxic intermediates accumulated in the treated solution, showing the advantage of the applied method. The obtained results suggested that the BPA oxidation (in terms of degradation and DOC mineralization) in the photo-Fenton-like system was driven by the conjugated radical chain mechanism. The latter includes, along with direct photolysis, photo-induced and catalytic processes involving in situ generated reactive oxygen species (^•^OH and SO_4_^−•^).

The identification of intermediates is crucial in determining the degradation pathway for a selected pollutant. An HPLC/MS analysis revealed the stepwise degradation to a number of intermediates. Specifically, the following products were identified after 5 min treatment in the UV/Fe^2+^/S_2_O_8_^2^^−^ system: monohydroxylated BPA (*m*/*z* = 243), dihydroxylated BPA (*m*/*z* = 259), hydroxybenzoaldehyde (*m*/*z* = 121), 3-hydroxy-4-methoxy benzoic acid (*m*/*z* = 152), 2.4 bis (1.1–dimethylethyl) phenol (*m*/*z* = 206), 4-isopropyl-benzene-1,2-diol (*m*/*z* = 164) and fumaric acid (m/z = 115). This is in agreement with the literature data on similar compounds, which were detected during BPA oxidation with persulfate activated with a low-pressure mercury lamp [29], ultrasonication [37] and thermal exposure [38].

**Figure 6:**
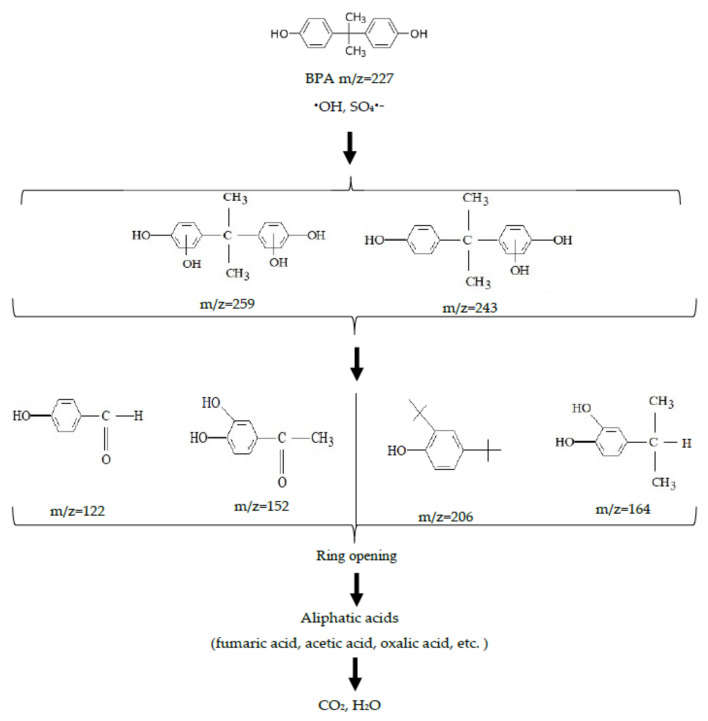
A proposed pathway of BPA oxidation in the UV/Fe^2+^/S_2_O_8_^2−^ system using a UV KrCl excilamp.

The mechanism of BPA decomposition under SO_4_^•−^ radicals attack, which was described previously [29], was likely to take place during the UV-enhanced Fenton-like process employed in this study. In the hybrid UV/Fe^2+^/S_2_O_8_^2−^ system, a high mineralization of dissolved organic matter (up to 84%) was achieved along with complete degradation. This was due to the fact that on attacking BPA by SO_4_^•−^ radicals, hydroxycyclohexadienyl cation radicals were formed due to direct electron transfer. These radicals underwent hydroxylation via the hydrolysis stage, resulting in the formation of mono- and dihydroxylated bisphenols. Upon further oxidation (after the breakdown of isopropyl group) hydroxybenzoaldehyde, 3-hydroxy-4-methoxy benzoic acid, 2.4 bis (1.1–dimethylethyl) phenol, 4-isopropyl-benzene-1.2-diol and other aromatic compounds with one benzene ring were formed. At the next stage, the aromatic ring opened with a formation of organic acids and their further mineralization took place. The following pathway of BPA oxidation in the UV/Fe^2+^/S_2_O_8_^2−^ system using the KrCl excilamp could be proposed (Figure 6).

## 4. Conclusions

The kinetics of BPA degradation and mineralization by photo-Fenton-like processes using a quasi-monochromatic UVC radiation of a KrCl excilamp (222 nm) were studied. It was found that the photo-Fenton-like system was the most efficient in the following order: Fe^2+^/S_2_O_8_^2−^ < UV < UV/S_2_O_8_^2−^ < UV/Fe^2+^/S_2_O_8_^2−^. A complete removal of the target compound and a high mineralization (84%) were achieved in the UV/Fe^2+^/S_2_O_8_^2−^ system at a molar ratio of [S_2_O_8_^2−^]:[BPA] = 5:1 without the accumulation of toxic products. A significant synergistic effect driven by the conjugated radical chain mechanism was found in this hybrid system. The primary intermediates of BPA degradation were identified and the corresponding oxidation pathway was proposed. The results suggested that a KrCl excilamp could be used as an alternative mercury-free UV source in photo-Fenton-like processes for an efficient mineralization and detoxification of organic micropollutants in low turbid water.

## Figures and Tables

**Table 1:** The summary of literature on the degradation of organic pollutants using UV excilamps: KrCl (222 nm), XeBr (283 nm), Cl_2_ (259 nm), XeCl (308 nm).

Photo Activation System	Pollutant, Initial Concentration, mg/L	Excilamp	Conditions	Removal, %	Rate Constant	Refs.
UV/Fe^2+^/S_2_O_8_^2^^−^	Atrazine, 4	KrCl	[Fe^2+^] = 89.5 μM, [S_2_O_8_^2^^−^] = 312 μM,	99	24 × 10^−2^ cm^2^/mJ	[17]
			Reaction time 5 min			
UV/S_2_O_8_^2^^−^	Atrazine, 4	KrCl	[S_2_O_8_^2^^−^] = 312 μM	NR *	13 × 10^−2^ cm^2^/mJ	
UV/H_2_O_2_	Methylene Blue, 18	KrCl XeCl	C(H_2_O_2_) = 0.05% *v*/*v*	99 30	1.99 min^−1^ 0.202 min^−1^	[18]
UV/Fe^2+^/S_2_O_8_^2^^−^	Orange III, 10	KrCl	[Fe^2+^] = 180 μM, [S_2_O_8_^2^^−^] = 180 μM, Reaction time 15 min	100	NR	[19]
UV/S_2_O_8_^2^^−^	Orange III, 10	KrCl	[S_2_O_8_^2^^−^] = 180 μM, Reaction time 15 min	70	NR	
UV/H_2_O_2_	Atrazine, 1 Triclosan, 1	KrCl	[H_2_O_2_] = 0.6 mM	NR	3.2 × 10^−2^ cm^2^/mJ 6.2 × 10^−2^ cm^2^/mJ	[20]
UV/Fe^2+^/H_2_O_2_	Methylene Blue, 25	KrCl	[Fe^2+^] = 2 mg/L, [H_2_O_2_] = 390 μM, Reaction time 10 min	100	0.12 min^−1^	[21]
UV/H_2_O_2_	2,4-dichloro-phenoxiacetic acid, 50	KrCl	[H_2_O_2_] = 452 μM, Reaction time 40 min	100	0.13 min^−1^	[13]
UV/H_2_O_2_	2-methyl–4-chlorophenol, 28.5	KrCl	[H_2_O_2_] = 2 mM, Reaction time 120 min	100	NR	[22]
UV/Fe^2+^/H_2_O_2_	Congo Red, 12.5	KrCl	[Fe^2+^] = 3 mg/L, [H_2_O_2_] = 1.3 mM, Reaction time 90 min	100	0.268 min^−1^	[23]
UV/H_2_O_2_	Para-chlorobenzoic acid, 25	KrCl, XeBr	[H_2_O_2_] = 4 mM	NR	0.13×10^−2^ cm^2^/mJ 0.10×10^−2^ cm^2^/mJ	[24]
UV/Fe^2+^/H_2_O_2_	4-chlorophenol, 50	KrCl	[Fe^2+^] = 5 mg/L, [H_2_O_2_] = 9.7 mM, Reaction time 5 min	100	NR	[25]
UV/H_2_O_2_	Congo Red, 100	KrCl, XeBr Cl_2_	[H_2_O_2_] = 7.2 mM, Reaction time 30 min	100 80 50	NR	[26]

* not reported.

**Table 2:** The effect of persulfate concentration (in terms of molar ratio of S_2_O_8_^2–^ and BPA) on the kinetics of BPA degradation in the UV/S_2_O_8_^2−^ system.

[S_2_O_8_^2−^]:[BPA], M/M	*k*, min^−1^	τ_1/2_, min	R^2^
-	0.03	23.11	0.997
3:1	0.17	4.03	0.992
5:1	0.25	2.82	0.991
10:1	0.47	1.49	0.959
20:1	0.78	0.89	0.981

**Table 3:** The toxicity indices (T) of aqueous solutions before and after treatment in the UV/Fe^2+^/S_2_O_8_^2^^−^ system. [BPA]_0_ = 43.8 µM, [S_2_O_8_^2^^−^]:[BPA] = 5: 1, [Fe^2+^] = 71.4 µM, pH_0_ = 5.7.

Sample	Before			After					
				5 min			30 min		
	Bioluminescence Intensity, imp/s		T	Bioluminescence Intensity, imp/s		T	Bioluminescence Intensity, imp/s		T
	Control	Sample		Control	Sample		Control	Sample	
1	21,484	14,582	30.3	22,586	19,490	13.7	22,544	23,905	0
2	20,233	16,971	16.1	22,943	20,666	9.9	21,104	24,311	0
3	19,117	16,275	14.8	19,217	18,003	6.3	20,006	28,033	0
Mean	20.4 ± 8.5			9.9 ± 3.7			0		

## References

[1] Kim J.-J., Kumar S., Kumar V., Lee Y.-M., Kim Y.-S., Kumar V. (2019). Bisphenols as a legacy pollutant, and their effects on organ vulnerability. Int. J. Environ. Res. Public Health.

[2] Gorini F., Bustaffa E., Coi A., Iervasi G., Bianchi F. (2020). Bisphenols as environmental triggers of thyroid dysfunction: Clues and evidence. Int. J. Environ. Res. Public Health.

[3] Ushani U., Lu X., Wang J., Zhang Z., Dai J., Tan Y., Wang S., Li W., Niu C., Cai T. (2020). Sulfate radicals-based advanced oxidation technology in various environmental remediation: A state-of-the-art review. Chem. Eng. J..

[4] Giannakis S., Lin K.-Y.A., Ghanbari F. (2021). A review of the recent advances on the treatment of industrial wastewaters by sulfate radical-based advanced oxidation processes (SR-AOPs). Chem. Eng. J..

[5] Yang J., Zhu M., Dionysiou D.D. (2021). What is the role of light in persulfate-based advanced oxidation for water treatment?. Water Res..

[6] Matzek L.W., Carter K.E. (2016). Activated persulfate for organic chemical degradation: A review. Chemosphere.

[7] Wacławek S., Lutze H.V., Grübel K., Padil V.V.T., Černík M., Dionysiou D.D. (2017). Chemistry of persulfates in water and wastewater treatment: A review. Chem. Eng. J..

[8] Wang J., Wang S. (2018). Activation of persulfate (PS) and peroxymonosulfate (PMS) and application for the degradation of emerging contaminants. Chem. Eng. J..

[9] Yang Q., Ma Y., Chen F., Yao F., Sun J., Wang S., Yi K., Hou L., Li X., Wang D. (2019). Recent advances in photo-activated sulfate radical-advanced oxidation process (SR-AOP) for refractory organic pollutants removal in water. Chem. Eng. J..

[10] Ismail L., Ferronato C., Fine L., Jaber F., Chovelon J.-M. (2017). Elimination of sulfaclozine from water with SO_4_^•−^ radicals: Evaluation of different persulfate activation methods. Appl. Catal. B Environ..

[11] Delavaran Shiraz A., Takdastan A., Borghei S.M. (2018). Photo-Fenton like degradation of catechol using persulfate activated by UV and ferrous ions: Influencing operational parameters and feasibility studies. J. Mol. Liq..

[12] Graça C., Correia de Velosa A., Teixeira A.C. (2015). Amicarbazone degradation by UVA-activated persulfate in the presence of hydrogen peroxide or Fe^2+^. Catal. Today.

[13] Murcia M.D., Vershinin N.O., Briantceva N., Gomez M., Gomez E., Cascales E., Hidalgo A.M. (2015). Development of a kinetic model for the UV/H_2_O_2_ photodegradation of 2,4-dichlorophenoxiacetic acid. Chem. Eng. J..

[14] Matafonova G., Batoev V. (2012). Recent progress on application of UV excilamps for degradation of organic pollutants and microbial inactivation. Chemosphere.

[15] Gomez M., Murcia M., Gomez J.L., Matafonova G., Batoev V., Christofi N. (2010). Testing a KrCl excilamp as new enhanced UV source for 4-chlorophenol degradation: Experimental results and kinetic model. Chem. Eng. Process. Process Intensif..

[16] Sosnin E., Avdeev S., Tarasenko V., Skakun V., Schitz D. (2015). KrCl barrier-discharge excilamps: Energy characteristics and applications (Review). Instrum. Exp. Tech..

[17] Popova S., Matafonova G., Batoev V. (2019). Simultaneous atrazine degradation and *E. coli* inactivation by UV/S_2_O_8_^2−^/Fe^2+^ process under KrCl excilamp (222 nm) irradiation. Ecotoxicol. Env. Saf..

[18] Aristizábal A., Perilla G., Lara-Borrero J.A., Diez R. (2020). KrCl and XeCl excilamps and LP-Hg lamp for UV and UV/H_2_O_2_ decolourization of dyes in water. Environ. Technol..

[19] Sizykh M., Batoeva A., Tsydenova O. (2018). UV-Activated persulfate oxidation of Orange III dye using KrCl excilamp. Clean Soil Air Water.

[20] Matafonova G., Batoev V. (2017). Comparison of energy requirements for removal of organic micropollutants from lake water and wastewater effluents by direct UV and UV/H_2_O_2_ using excilamp. Desal. Water Treat..

[21] Gómez M., Murcia M.D., Gómez E., Ortega S., Sánchez A., Thaikovskaya O., Briantceva N. (2016). Modelling and experimental checking of the influence of substrate concentration on the first order kinetic constant in photo-processes. J. Environ. Manag..

[22] Tchaikovskaya O., Karetnikova E., Murcia M., Gomez M., Gómez J.L. (2014). Photodegradation of 2-methyl-4-chlorophenol in a KrCl exciplex flow-through photoreactor: A kinetic study. Desalination Water Treat..

[23] Murcia M., Gomez M., Ortega-Requena S., Hidalgo A., Gomez E., Gómez J. (2015). Degradation of congo red in a exciplex flow-through photoreactor. Kinetic study. Afinidad Barc..

[24] Matafonova G.G., Batoev V.B. (2012). Comparison of UV and UV/H_2_O_2_ treatments using excilamps for removal of monochlorophenols in the molecular and anionic form. J. Environ. Sci. Health Part A.

[25] Gomez M., Murcia M.D., Gomez J.L., Gomez E., Maximo M.F., Garcia A. (2012). A KrCl exciplex flow-through photoreactor for degrading 4-chlorophenol: Experimental and modelling. Appl. Catal. B Environ..

[26] Murcia M., Gomez M., Gomez E., Gómez J., Christofi N. (2011). Photodegradation of congo red using XeBr, KrCl and Cl_2_ barrier discharge excilamps: A kinetics study. Desalination.

[27] Canonica S., Meunier L., von Gunten U. (2008). Phototransformation of selected pharmaceuticals during UV treatment of drinking water. Water Res..

[28] Sorokina E.V., Yudina T.P., Bubnov I.A., Danilov V.S. (2013). Assessment of iron toxicity using a luminescent bacterial test with an *Escherichia coli* recombinant strain. Microbiology.

[29] Sharma J., Mishra I.M., Kumar V. (2016). Mechanistic study of photo-oxidation of Bisphenol-A (BPA) with hydrogen peroxide (H_2_O_2_) and sodium persulfate (SPS). J. Environ. Manag..

[30] Leitner N.K.V., Stefan M.I. (2018). Sulfate radical Ion—Based AOPs. Advanced Oxidation Processes for Water Treatment.

[31] Huang W., Bianco A., Brigante M., Mailhot G. (2018). UVA-UVB activation of hydrogen peroxide and persulfate for advanced oxidation processes: Efficiency, mechanism and effect of various water constituents. J. Hazard. Mater..

[32] Grčić I., Vujević D., Koprivanac N. (2010). Modeling the mineralization and discoloration in colored systems by (US)Fe^2+^/H_2_O_2_/S_2_O_8_^2−^ processes: A proposed degradation pathway. Chem. Eng. J..

[33] Kusic H., Peternel I., Ukic S., Koprivanac N., Bolanca T., Papic S., Bozic A.L. (2011). Modeling of iron activated persulfate oxidation treating reactive azo dye in water matrix. Chem. Eng. J..

[34] Bu L., Shi Z., Zhou S. (2016). Modeling of Fe(II)-activated persulfate oxidation using atrazine as a target contaminant. Sep. Purif. Technol..

[35] Wu L., Zhang Q., Hong J., Dong Z., Wang J. (2019). Degradation of bisphenol A by persulfate activation via oxygen vacancy-rich CoFe_2_O_4–x_. Chemosphere.

[36] Serra-Pérez E., Álvarez-Torrellas S., Ismael Águeda V., Delgado J.A., Ovejero G., García J. (2019). Insights into the removal of Bisphenol A by catalytic wet air oxidation upon carbon nanospheres-based catalysts: Key operating parameters, degradation intermediates and reaction pathway. Appl. Surf. Sci..

[37] Darsinou B., Frontistis Z., Antonopoulou M., Konstantinou I., Mantzavinos D. (2015). Sono-Activated persulfate oxidation of bisphenol A: Kinetics, pathways and the controversial role of temperature. Chem. Eng. J..

[38] Potakis N., Frontistis Z., Antonopoulou M., Konstantinou I., Mantzavinos D. (2017). Oxidation of Bisphenol A in water by heat-activated persulfate. J. Environ. Manag..

